# Left atrial voltage mapping: defining and targeting the atrial fibrillation substrate

**DOI:** 10.1007/s10840-019-00537-8

**Published:** 2019-05-10

**Authors:** Iain Sim, Martin Bishop, Mark O’Neill, Steven E. Williams

**Affiliations:** Division of Imaging Sciences and Biomedical Engineering, King’s College London, 4th Floor North Wing, St. Thomas’ Hospital, 249 Westminster Bridge Road, London, SE1 7EH UK

**Keywords:** Bipolar voltage, Atrial fibrillation, Atrial ablation, Atrial fibrosis

## Abstract

Low atrial endocardial bipolar voltage, measured during catheter ablation for atrial fibrillation (AF), is a commonly used surrogate marker for the presence of atrial fibrosis. Low voltage shows many useful associations with clinical outcomes, comorbidities and has links to trigger sites for AF. Several contemporary trials have shown promise in targeting low voltage areas as the substrate for AF ablation; however, the results have been mixed. In order to understand these results, a thorough understanding of voltage mapping techniques, the relationship between low voltage and the pathophysiology of AF, as well as the inherent limitations in voltage measurement are needed. Two key questions must be answered in order to optimally apply voltage mapping as the road map for ablation. First, are the inherent limitations of voltage mapping small enough as to be ignored when targeting specific tissue based on voltage? Second, can conventional criteria, using a binary threshold for voltage amplitude, truly define the extent of the atrial fibrotic substrate? Here, we review the latest clinical evidence with regard to voltage-based ablation procedures before analysing the utility and limitations of voltage mapping. Finally, we discuss omnipole mapping and dynamic voltage attenuation as two possible approaches to resolving these issues.

## Introduction

Designing effective ablation strategies for persistent atrial fibrillation remains a major challenge in cardiac electrophysiology. Since the development of the pulmonary vein isolation procedure over 20 years ago, several additional ablation strategies have been proposed, including stepwise ablation, complex electrogram ablation and rotor/focal impulse ablation. Although these techniques showed promise in single-centre studies, converting this success into widespread improvements in procedural outcome in multiple centres has remained challenging. Nevertheless, emerging evidence suggests that achieving sinus rhythm could result in significant improvements in both morbidity and mortality for AF patients, and as such new approaches to AF ablation are sought.

**Fig. 1:**
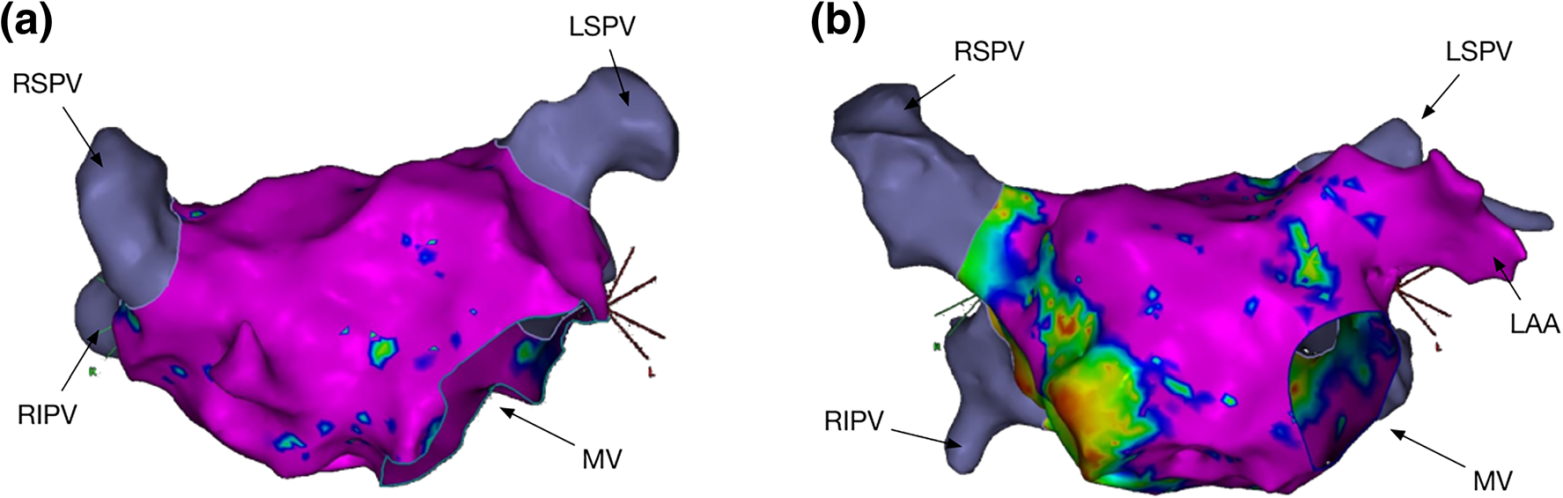
Left atrial voltage mapping. Two examples of high density left atrial voltage maps created during AF ablation are shown using CARTO (Biosense Webster, Diamond Bar, USA) and recorded using a LassoNav catheter. Panel (**a**) includes 1122 points. Panel (**b**) includes 1489 points. In both cases, purple colour represents electrogram voltage > 0.5 mV. In (**a**), minimal/no LVA are present whereas in (**b**), there are areas of low voltage affecting the LA septum and posterior wall

Recently, left atrial bipolar endocardial voltage mapping (Fig. 1) has emerged as an invasive tool used during radiofrequency ablation procedures for defining the AF substrate. Contemporary electro-anatomic mapping platforms used during AF ablation allow hundreds to thousands of voltage points to be mapped onto a geometric model of the atrial endocardium. It is proposed that low atrial bipolar voltage amplitude is a surrogate marker for the presence of native atrial fibrosis and that atrial fibrosis plays a key role in maintaining AF. As such, several clinical studies have described approaches for isolating areas of left atrial low voltage, showing promise as methods to reduce arrhythmia recurrence after AF ablation. However, the methodology for defining low voltage areas has not been standardised and a clear voltage threshold for abnormality has never been histologically validated. These observations, and the potential for widespread clinical use of these new treatment strategies, necessitate a thorough understanding of the techniques, benefits and challenges of using voltage mapping to define and target the AF substrate.

In this review article, we first summarise the contemporary studies of voltage-guided ablation. Recognising the variable outcomes from these clinical studies, we then examine the technical aspects of recording the extracellular electrical field potential (voltage) during clinical procedures, and the relationship between these signals and recognised components of the AF substrate. Subsequently, we present the available data defining normal/abnormal atrial voltage and the evidence directly linking voltage thresholds to atrial fibrosis in humans. Finally, we discuss recent technical developments that may overcome some of the limitations of bipolar voltage mapping for defining the AF substrate.

## Clinical studies of voltage-guided ablation

Rolf et al. provided the first description of atrial low voltage area ablation for AF [1]. In their non-randomised study, 178 patients underwent pulmonary vein isolation plus additional isolation of low voltage areas. Compared to an historical control group of 26 patients with low voltage areas undergoing pulmonary vein isolation alone, patients receiving voltage-guided ablation had significantly greater arrhythmia-free survival. Subsequently, seven further observational studies have compared outcomes with low voltage area-based ablation with standard ablation [2–7]. Broadly, these studies fall into two categories: those comparing standard ablation to low voltage-guided ablation in the subset of patients with atrial low voltage, and those comparing ablation strategies (standard ablation vs. standard ablation plus low voltage-guided ablation) in unselected patients undergoing AF ablation (Table 1). Although these studies demonstrate a variable impact of voltage-guided ablation on arrhythmia recurrence, a recent meta-analysis has shown an overall improvement in outcomes with low voltage area-guided ablation [8].

**Table 1:** Summary of trial outcomes of substrate-based ablation

	Rhythm during mapping	Mapping catheter (electrode size)	Mapping density (mean ± SD points per map)	Control group *N* (PAF, PsAF)	Study group *N* (PAF, PsAF)	Control intervention	LVA intervention	F/U (months)	Arrhythmia-free survival (control vs. study)	Additional electrogram features?
Schreiber 2017 [5]	Sinus rhythm	Ablation (3.5 mm)	> 100	No LVA *n* = 49 (39, 10)	< 0.5 mV *n* = 92 (34, 58)	PVI alone	PVI + BIFA + L	12	84% vs. 69% *P* = 0.16	None
Yagishita 2017 [9]	AF	Ablation (3.5 mm)	166 ± 62	No LVA *n* = 42 (15, 27)	< 0.5 mV *n* = 159 (22, 137)	PVI alone	PVI + HI	12	71% vs. 72% *P* = 0.746	None
Yamaguchi 2016 [6]	Sinus rhythm	Ablation and mapping (4 mm and 1 mm)	576 ± 150	No LVA *n* = 62 (15, 47)	<0.5mV *n* = 39 (18, 21)	PVI alone	PVI + HI	32	79% vs. 72% *P* = 0.400	None
Jadidi 2016 [3]	AF	Mapping (1 mm)	1024 ± 124	Unselected *n* = 66 (0, 66)	Unselected *n* = 85 (0, 85)	PVI alone	PVI + H + RA (further ablation only to terminate AF)	13	47% vs. 69% *P* < 0.001	< 0.5 mV, fractionation, rotational activity, rapid local activation
Yang 2016 [7]	Sinus rhythm	Mapping (1 mm)	628 ± 212	Unselected *n* = 78 (0, 78)	Unselected *n* = 86 (0, 86)	Stepwise	PVI + HI	30	51% vs. 70% *P* = 0.011	< 1.3 mV, > 50 ms, ≥ 3 deflections
Cutler 2016† [4]	Sinus rhythm	Ablation (not stated)	Not stated	Unselected *n* = 76 (0, 76)	Unselected *n* = 65 (0, 65)	PVI + PWI (discretionary)	PVI + PWI	12	57% vs. 80% *P* = 0.005	None
Kottkamp 2016 [2]	Sinus rhythm	Ablation (4 mm)	100–120	No LVA *n* = 13 (0, 13)	< 0.5 mV *n* = 18 (0, 18)	PVI alone	PVI + BIFA	12	69% vs. 72% *P* = 0.742	None
Rolf 2014 [1]	Sinus rhythm	Mapping and ablation (2.1 and 4 mm)	115 ± 35	No LVA *n* = 131 (56, 75) With LVA *n* = 26 (9, 17)	<0.5mv *n* = 47 (6, 41)	PVI alone	PVI + HL	12	62% vs. 27% vs. 70%	None

References are highlighted within the table

PVI, pulmonary vein isolation; H, homogenisation; I, isthmus; L, linear; T, transition zone; LVA, low voltage areas; PWI, posterior wall isolation; BIFA, box isolation of fibrotic area; RA, right atrium

However, there was significant heterogeneity in mapping strategies, patient selection and low voltage area prevalence between these studies and therefore direct comparisons are difficult to make. In particular, rhythm during mapping, electrode size and mapping resolution all varied between studies. For example, Yagishita et al. reported a much higher prevalence of low voltage areas (79%) than other studies, perhaps in part due to mapping in AF [9]. Similarly, Jadidi et al. performed voltage mapping in AF but ablation in sinus rhythm, which led to difficulty in the detection of low voltage areas once sinus rhythm had been restored [3]. Schreiber et al.’s study showed an overall benefit to low voltage ablation, but did not show improvement for those patients with the most extensive low voltage areas, in whom outcomes were similar to those who underwent conventional ablation with pulmonary vein isolation for persistent AF (40.9% arrhythmia free at 1 year) [5], suggesting that a voltage-based strategy may not be appropriate for all patients.

**Fig. 2:**
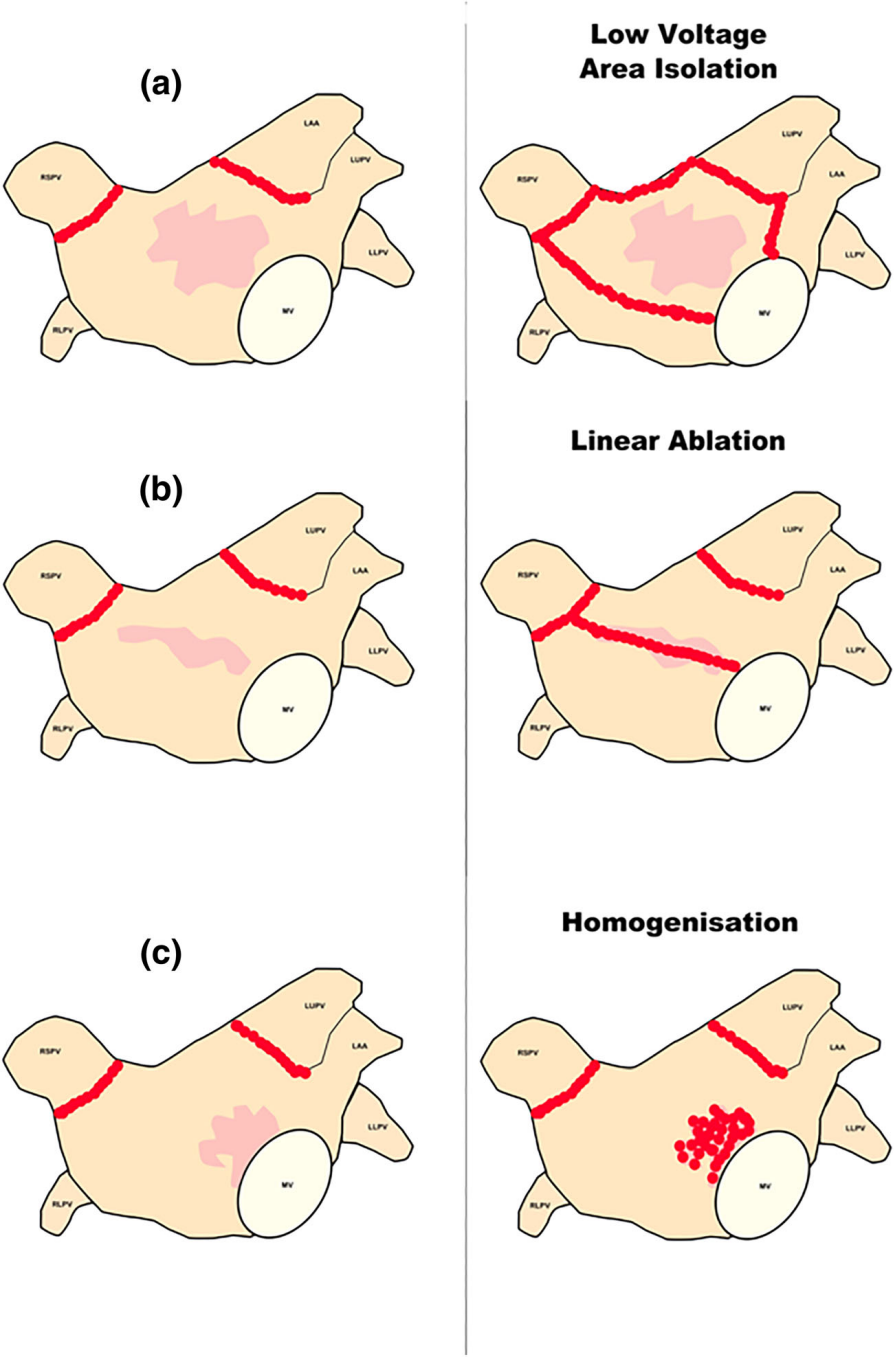
Approaches to low voltage area ablation. Strategies described by Kircher et al. [5] to individualise substrate-based ablation treatment. (**a**) Low voltage isolation strategy for treatment of low voltage areas, shown in light red. (**b**) Linear ablation across a low voltage area. (**c**) Homogenisation of a low voltage area

**Fig. 3:**
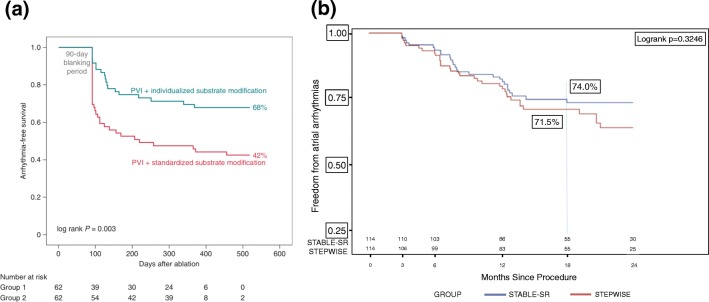
Clinical outcomes of randomised studies comparing voltage-guided ablation to standard ablation. (**a**) Kaplan–Meier curves comparing individualised versus standardised substrate modification, showing arrhythmia free survival and demonstrating a statistically significant benefit for an individualised approach [10]. (**b**) Kaplan–Meier curves from the STABLE SR trial comparing stepwise treatment for AF with substrate-based ablation using voltage mapping, showing no evidence of benefit [11]. The figures were adapted with permission

More recently, two randomised controlled clinical trials of voltage-informed intervention have been performed, with contrasting outcomes [10, 11]. Kircher et al. allocated a mixed group of AF patients to either low voltage area isolation (Fig. 2) or to standard treatment and showed significantly increased arrhythmia-free survival in the low voltage area isolation group at 1 year (68% vs. 42%, log rank *P* = 0.003) [10] (Fig. 3a). However, a low success rate for persistent AF patients in the control group may have contributed in part to the difference between these groups. In contrast, Yang et al. [11] randomised 229 patients with non-paroxysmal AF to either low voltage area-guided ablation, with additional complex electrogram ablation, or standard stepwise ablation including linear ablation but did not show a significant improvement in outcome (74% vs. 71%, *P* = 0.325) (Fig. 3b). However, procedure times were significantly shorter with significantly less ablation and shorter fluoroscopy times using the low voltage area-based approach.

Given the conflicting results of the clinical trials, two important questions arise. First, does bipolar voltage mapping actually provide a ‘gold standard’ definition of the atrial substrate and, second, under what conditions, with what techniques and with what thresholds should voltage mapping be performed? To answer these questions, it is necessary to first examine the technical aspects of bipolar voltage mapping.

## Technical aspects of voltage mapping

**Fig. 4:**
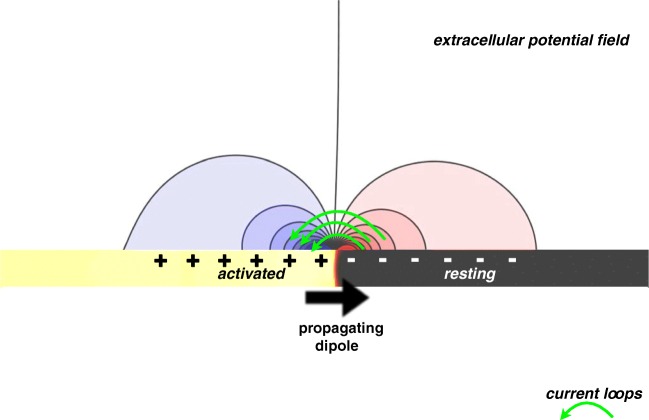
Extracellular field created by an activation wave-front. At the exact region of the wave-front, axial current flows from the activated tissue to the resting tissue, creating a system representative of a propagating dipole. Transmembrane current then flows out of the membrane ahead of the wave, to return at the wave-back via newly activated sodium channels, thus forming a current loop. Consequently, the extracellular field is positive ahead of the wave-front and negative behind it

Unipolar signals are generated by recording the extracellular potential (*φ*_*e*_) at a particular recording location in close proximity, or ideally in contact with, the endocardial surface of the atrium. The local extracellular potential near the surface is changed in response to an electrical activation wave propagating through the cardiac tissue beneath it. Such a wave is commonly thought of as a pair of propagating dipoles, one representing the depolarising activation wave-front and the other representing the repolarising wave-back (Fig. 4).

Ahead of the wave-front, the cardiac intracellular potential is more negative than the cardiac extracellular potential (*φ*_*i*_ < *φ*_*e*_), leading to a negative resting membrane potential, *V*_*m*_, of approximately −80 mV. This potential gradient drives a flow of intracellular axial current through the gap junctions activating the neighbouring downstream cell by an outward positive capacitive current which begins to increase *V*_*m*_ (i.e. causing depolarisation). As *V*_*m*_ rises, sodium channels become activated, leading to a large inward sodium current behind the wavefront which further increases *V*_*m*_ by an inward flux of positive (Na^+^) ions causing *φ*_*i*_ to become positive with respect to *φ*_*e*_.

The net effect is a biphasic (positive followed by negative) membrane current which spatially is seen as a ‘current loop’. The intracellular space remains positive with respect to the extracellular spacing during the action potential plateau (with a *V*_*m*_ during the plateau of approximately 0–20 mV). Thus, this spatial region of transition from negative to positive at the wave-front can be represented by a positive dipole, pointing in the same direction as wave propagation.

It can be shown mathematically that the extracellular potential recorded at a particular location due to a propagating dipole depends on

1. The strength of the dipole source.
2. The respective angle the propagating dipole makes with the recording site.
3. The distance separating the dipole and the recording site.

A positive dipole, propagating in the direction of the wave, will generate a positive signal recorded by an electrode at a location as the dipole approaches the recording site, corresponding to the positive potential ahead of the wave-front. The recorded signal will then switch to a negative signal as the dipole propagates away from the recording site, corresponding to the negative potential behind the wave-front. When the dipole passes exactly in line with the recording site, the signal is zero. These basic biophysical phenomena, as recorded at the cellular level, describe the shape of unipolar electrograms recorded from the surface of the atria by contemporary mapping catheters.

Bipolar signals are taken as the difference between two neighbouring unipolar signals, either with the use of a differential amplifier or via post-processing of unipolar signals. Extra-cardiac signals, i.e. noise and far-field signals from adjacent cardiac structures, will be similar between the two signals and are thus removed, or minimised, by these subtractions. However, as the wave-front will be at a different distance from the two unipolar recording sites, there will be a temporal offset between the two unipolar signals, depending on exactly when the wave-front passes beneath the recording site. This difference is reflected in the bipolar electrogram morphology.

Voltage signals recorded from individual electrodes are converted by contemporary electro-anatomic systems into colour-coded voltage maps, providing a static representation of time-dependent electrical activation of the atrium. As such, a recording window must be specified for analysis. In the case of regular atrial rhythms, this window of interest is set around a fixed temporal reference, usually set to include a single cycle of activation. In the case of irregular rhythms (i.e. AF), the window of interest is set as a duration (e.g. 6–30 s) and is independent of activation of other parts of the chamber. Voltage amplitude is then defined as the maximal peak-to-peak voltage within the window of interest. It is important to recognise the raw data constituting a voltage map consists of three-dimensional Cartesian co-ordinates of the electrode positions at the time of recording together with a single number for each position representing the voltage. These data are transformed, typically in real time, into a voltage map by two processes: projecting the recording co-ordinates onto the atrial shell and interpolating the voltage data across the surface of the shell. To the best of our knowledge, the proprietary algorithms used for projection and interpolation are not publicly available, though the operator is generally able to define the degree of interpolation between voltage points. To define low voltage areas, the final step requires the user to set a threshold defining low voltage, typically less than 0.5 mV for atrial mapping [12–14].

Based on the above principles, several non-substrate factors (Table 2) can theoretically influence electrogram voltage, some of which have been demonstrated during *in vivo* mapping.

**Table 2:** Non-substrate-based effects upon bipolar voltage (references are shown within the table)

Factor	Direction of effect on electrogram amplitude (theoretical/pre-clinical)	Direction of effect (clinical)	Clinical demonstration of effect	Reference
Non-parallel activation direction	Decrease	–	No	[17, 82]
Increasing angle of incidence	30° improves lesion diagnosis accuracy	–	No	[86]
Increasing electrode size	Variable dependent on tissue	Variable dependent on tissue	Yes	[19, 20]
Increasing electrode spacing	Increase	Increase	Yes	[17, 21]
Increasing tissue contact force	Increase	Increase at low contact force only	Yes	[23]
Bandpass filtering	Effect is based on which filter is applied	Increasing frequency of high pass reduces amplitude	Yes*	[24]

*Only for non-contact unipolar mapping

### Activation direction

The relationship between the orientation of the recording bipole and the wave-front dipole will influence the arrival time of the activating wave-front at each electrode. Therefore, hypothetically, orienting the recording bipole exactly parallel to the wave-front creates identical electrograms on each unipole electrode and the resulting bipolar voltage is zero. Direction-dependent effects were previously demonstrated for much larger ventricular signals [15] but have also recently been demonstrated by comparing sinus rhythm/high right atrial pacing to coronary sinus pacing in the atrium. In contrast to predictions from simulated data, these effects are highly variable indicating that voltage change could not be used, for example, to infer activation direction [16].

### Electrode spacing

For a fixed conduction velocity, the distance separating the bipole electrodes determines the temporal offset between the wave-front arrival time at each electrode. For close separations (and/or high conduction velocities), the unipolar waveforms will be almost aligned; for large separations (and/or low conduction velocities), they will be separated in time. The exact temporal offset thus determines the amplitude and morphology of the bipolar signal. In computer simulation studies, increasing interelectrode distance leads to increasing voltage which plateaus in healthy, but not diseased, tissue with spacing greater than 4 mm [17]. Further, the contribution of farfield signal may be greater when electrodes are more widely spaced [18].

### Electrode size

Electrodes covering a larger surface area have been shown to record larger amplitude signals [19], while in others smaller electrodes on mapping catheters generated statistically significant increases in mean amplitude [20]. The characteristics of the underlying atrial tissue are likely to modulate these effects. For example, in one study, the presence of fibrosis, larger electrodes may summate voltages from both healthy and fibrotic areas and therefore record lower, not higher, amplitude signals [21].

Considering the above effects, the specifications of individual catheters are clearly important for interpreting bipolar voltage signals (Table 3) especially given that it appears size and spacing of electrodes have different contributions to the overall voltage [22]. The wide array of electrode sizes and spacings used in clinical studies and the lack of direct comparisons between mapping catheters prevents a sound appraisal of the effect electrode size and spacing has on the identification of low voltage areas in clinical studies at present. However, there appear to be different effects depending on whether it is healthy or fibrotic tissue being measured.

**Table 3:** Studies comparing catheters with various electrode sizes and spacing

Catheter tested			Comparator catheter			Effect on overall voltage	Effect on LVA size	Reference
Name	Electrode size (mm^2^)	Electrode spacing (mm)	Name	Electrode size (mm^2^)	Electrode spacing (mm)			
Pentaray	1	2–6	Thermocool	3.5	1–6–2	Increase in scar only	Decrease	[21]
Lasso	1	8	Thermocool	3.5	1–6–2	Increase	Decrease	[20]
Orion Basket	0.4	2.5	Pentaray	1	2–6	–	Unchanged	[87]
			Lasso	1	1–2			
Intella Tip	0.8 (within 4.5 mm tip)	1.2	TactiCath	3.5	2–5–2	–	Increased	[88]
			Circular mapping catheter	1	5	–	Unchanged	
Inquiry Optima	1	7	Cool Flex	4	0.5–5–2	–	Decrease	[89]

Catheters studied: Thermocool SmartTouch CF contact force-sensing catheter (Biosense Webster); Pentaray (Biosense Webster); Variable Loop Eco Nav (Biosense Webster); Orion Basket catheter (Boston Scientific); Inquiry Optima (St. Jude Medical); Intella Tip Micro Fidelity MiFi O/I (Boston Scientific); TactiCath Quartz (St. Jude Medical); Cool Flex (IBI/St. Jude Medical); HD-Grid catheter (Abbott, St. Paul, Minnesota). Spacing is as quoted by the manufacturer and is not ‘centre to centre’ spacing

### Tissue contact

Although adequate and consistent contact with myocardium is required to record reliable voltage signals, the effect of ever-increasing contact force is less certain. For example, one study assessing voltages recorded with a contact force sensing catheter found a weak correlation between force and voltage but only at very low contact forces [23]. Once contact force was increased above 5*g* (0.05 N), there was no further increase in voltage.

### Filtering

Bandpass filtering may change the amplitude of any given electrogram, and thus the specific filter settings used may consequently alter the peak-to-peak amplitude of the recorded bipolar signal. During clinical mapping, bipolar electrograms are typically bandpass filtered with a high pass of 1–30 Hz and a low pass of 300–500 Hz with a notch filter at 50–60 Hz. To our knowledge, the direct relationship between filter settings and voltage amplitude for contact mapping has not been quantified. However, using non-contact mapping, Lin et al. showed that increasing the high-pass cut-off from 0.5 to 32 Hz reduced mean pre-ablation unipolar voltage amplitude from 0.93 ± 0.45 mV to 0.56 ± 0.47 mV [24].

We suggest that a consensus on the ideal voltage mapping strategy (electrode size, spacing, point density and filter settings) could contribute significantly to minimising variation between studies. Given these limitations, the next section will discuss the available evidence demonstrating the extent to which voltage mapping, in its present form, identifies a substrate for AF.

## Low voltage: a marker of the AF substrate?

A major challenge in evaluating the diagnostic performance of voltage mapping for identifying the AF substrate is the lack of a clear consensus on the form of that substrate. Indeed, the term ‘AF substrate’ has been used variably to refer to progressive structural alterations in atrial myocardium including bi-atrial enlargement, reduced atrial emptying function, myocyte hypertrophy, loss of myofibrils, accumulation of glycogen, and changes in mitochondrial shape and size [25–27]. Against this background, low voltage is most commonly considered a marker of the presence of atrial fibrosis and an associated fibrotic atrial cardiomyopathy [28] based on both direct (cardiac magnetic resonance imaging, serum markers) and indirect (atrial size/function and conduction velocity) evidence.

### Atrial fibrosis

Atrial fibrosis has been identified histologically in patients with AF [29] and patients with risk factors for AF [30, 31]. However, histological validation between low voltage and native atrial fibrosis is currently lacking. Cardiac magnetic resonance remains the only available technique for non-invasive assessment of atrial fibrosis. Late gadolinium enhancement has been correlated with atrial fibrosis by histological assessment in a small number of patients [32] and several studies have compared bipolar voltage with late gadolinium enhancement. Spragg et al. found that areas of scar identified by late enhancement imaging show lower voltage compared to non-scar regions (0.39 ± 0.61 mV vs. 1.38 ± 1.23 mV, *P* < 0.001) and that late gadolinium enhancement detects areas of low voltage (< 0.5 mV) with a sensitivity of 84% but with specificity of 68% [33]. Another study of 21 patients with paroxysmal AF correlated increased signal intensity with progressively lower bipolar voltage [34]. Not all studies demonstrated such a clear relationship. For example, in a study of 18 patients with persistent AF, Jadidi et al. showed that dense late gadolinium enhancement was associated with only a slightly lower mean voltage than non-dense late enhancement regions (0.6± 0.8 mV vs. 0.86± 0.89 mV, *P* < 0.001) [35]. However, more recently, Khurram et al. used the image intensity ratio (IIR, ratio of the atrial wall signal intensity to mean blood pool signal intensity) and found IIR to be strongly correlated with bipolar voltage: IIRs of 0.97 and 1.61 correlated with voltages of 0.5 mV and < 0.1 mV, respectively [36].

### Atrial size and function

Increasing atrial size is associated with lower mean atrial voltages, suggesting a link between atrial wall stress and morphological changes [37]. There is evidence from invasive and echocardiographic measurements that atrial pressure inversely correlates with mean atrial voltage [38] and in a study of 20 patients, Hunter et al. found that sites of higher wall stress displayed lower bipolar amplitudes [39]. Additionally, acute atrial dilatation has also been shown to increase the prevalence of low voltage zones, especially in the posterior wall [40], suggesting an alternative mechanism than the presence of fibrosis.

Echocardiographic indices of left atrial mechanical function, including left atrial ejection fraction, trans-mitral ‘A’ wave velocity-time integral [41] and synchrony using 3D speckle tracking [42], have all shown associations with voltage. Further, vortex flow analysis, representing pulsatility within the left atrium, inversely correlates with mean voltages obtained during left atrial pacing [43] and lower atrial voltage correlates with lower total left atrial strain (*r* = 0.71) as a measure of poor left atrial reservoir function [44].

### Conduction velocity

Atrial fibrosis influences local conduction velocity by forming ‘zig-zag’ like conduction paths, influencing anisotropy and by direct fibroblast–cardiomyocyte coupling resulting in an increased passive electric load to the cardiomyocytes [45]. Therefore, low conduction velocity is likely to coexist with low voltage areas. Indeed, total activation time is greater in patients with persistent AF compared to those with paroxysmal AF and correlates inversely with mean left atrial voltage [46]. Furthermore, patients with AF and low voltage areas (<0.5 mV) display reduced mean conduction velocity and local conduction delay through areas of low voltage. Similarly, right atrial low voltage is correlated with reduced conduction velocity (*r* = 0.65) [47]. However, in one study of AF patients with a minimum voltage of greater than 0.5 mV, conduction velocity was not significantly reduced [48].

### AF triggers

A number of studies have hypothesised that low voltage areas may identify trigger regions for AF by comparing voltage amplitude to high dominant frequency, complex fractionated electrograms and rotors. Patients with AF triggers from the pulmonary veins have lower voltage in the pulmonary vein antra and similarly patients with AF triggers in the superior vena cava show reduced voltage in the right atrium [49]. However, voltage is not significantly lower at specific areas with increased dominant frequency [50], and it has been demonstrated that the majority of high dominant frequency sites in AF do not correlate with low voltage areas recorded in sinus rhythm [51]. Conversely, a study using basket catheters found that voltages were significantly lower at high dominant frequency sites compared to non-high dominant frequency sites, but this only held for voltages measured during AF and not during sinus rhythm [52].

Complex fractionated electrogram sites in AF have previously been reported to represent critical sites for AF initiation and maintenance, but their relationship to low voltage areas in sinus rhythm is complex. A small study of persistent AF patients showed only a small overlap between complex fractionated electrogram sites and low voltage areas [14]. Further, when recording in AF, sites of complex fractionated electrogram sites are associated with normal bipolar voltage in sinus rhythm [51]. Indeed, voltages may be higher at complex fractionated electrogram sites than non-fractionated when recorded in sinus rhythm [53]. Narayan et al. found that only those complex fractionated electrogram sites with short cycle length (~ 150 ms) and narrow spectral DF were associated with low voltage, suggesting a link between lower voltage and rapid activation of atrial tissue [54]. Given these conflicting observations and varied possible mechanisms for the genesis of complex fractionated electrograms, it is challenging to identify a direct relationship between low voltage areas and electrogram fractionation.

Rotors, representing stable but meandering spiral waves, can anchor to areas of anatomic discontinuity such as fibrosis [55] and therefore voltage mapping may reveal sites important to maintaining atrial rotational activity. However, focal sources have variable voltage characteristics, and many areas displaying rotor sources do not show a reduction in voltage [56]. In one study, mapping showed a poor correlation between rotational activity and low voltage areas, with only half of rotors showing at least one electrogram with low voltage [57]. Schade et al. showed that in patients with persistent AF, 37% of ‘FIRM’ left atrial sources were located within low voltage areas and 22% were adjacent to low voltage areas. However, of those left atrial sources which resulted in successful ablation, 60% were entirely remote from low voltage areas. Further, it would be expected that if low voltage areas are able to support rotational activity, then there would be a relationship between the size of low voltage areas and the number of left atrial ‘FIRM’ sources, which was not demonstrated [58]. It remains unclear how low voltage areas and rotors interact or whether the formation of rotational activity is dependent on the presence of remote or local areas of fibrosis which could be detectable by voltage mapping.

## Defining low voltage areas

### Defining abnormal atrial tissue

In the studies discussed above, the relationship between clinical outcomes, AF substrate factors, AF triggers and low voltage areas depend critically on the voltage thresholds chosen to define low voltage. Histological correlation between low voltage and ventricular scar has been demonstrated in a porcine ventricular infarct model [59], and we have previously shown a direct relationship between low voltage and ablation scar in a porcine right atrial ablation model [60]. However, pathology data relating native, pre-ablation atrial fibrosis to atrial voltage mapping thresholds are currently unavailable. Therefore, in place of a histological definition, most contemporary studies define scar voltage amplitude of < 0.05 mV, a value which initially arose from the baseline noise of early electroanatomic mapping systems [61]. Additionally, a value of 0.5 mV is commonly used to define low voltage which, although convenient, is not based on the presence of defined underlying abnormalities in atrial structure or function.

Several studies have adopted a statistical approach to define abnormal voltage. These studies typically define abnormal as the voltage at the 5th centile of all mapping points. Kapa et al. reported voltages in 20 patients with paroxysmal AF, although only 10 patients had not undergone prior ablation. Using the statistical approach, they defined thresholds for atrial scar of < 0.2 mV for the posterior wall and pulmonary vein/left atrial junctions and < 0.45 mV for all other atrial segments [62]. In a related study, Lin et al. compared voltages of patients undergoing left-sided accessory pathway ablation with AF patients in sinus rhythm. In patients without AF, 95% of all electrograms were above 0.38 mV. They therefore defined low voltage areas as areas with voltage measured at contiguous points below 0.4 mV. However, 5% of electrograms in patients with persistent/long-standing AF showed voltages below 0.1 mV, which they therefore defined as ‘dense scar’ [63].

Other studies in supra-ventricular tachycardia patients undergoing left atrial mapping have identified different voltage thresholds for healthy tissue. For example, using the same 5th centile approach, Saghy et al. identified a bipolar voltage threshold of 0.5 mV in nine patients [12], while Yagashita et al. identified a threshold of 1.17 mV in six patients. Applying this latter threshold to AF patients, the same authors found 43% had voltages in the range 0.5–1.17 mV and that these patients were at increased risk of arrhythmia recurrence [64]. It is possible, therefore, that the standard threshold of < 0.5 mV is insensitive to atrial abnormalities important to arrhythmogenesis. Furthermore, these data suggest that there may not be a discrete absolute voltage threshold for atrial fibrosis and instead progressively lower voltages may be found with progressively increasing extents of atrial structural remodelling. Indeed, there is a significant body of evidence showing that progressively reducing voltages are seen as AF progresses from paroxysmal to persistent states. Teh et al. showed that mean left atrial voltage progressively decreased between patients with supraventricular tachycardia (2.8 mV), paroxysmal AF (2.2 mV) and persistent AF (1.8 mV) [65]. Similarly, Jadidi et al. showed mean voltages of 0.6 mV and 1.12 mV in patients with persistent and paroxysmal AF, respectively [66]. Further, Fiala et al. demonstrated a median voltage in patients with persistent AF of 0.41 mV compared with 0.99 mV in paroxysmal AF patients, although this mapping was undertaken during AF.

Though the figure of 0.5 mV is widespread throughout the literature, there is a need for pathological validation of voltage thresholds, or indeed demonstration that a true threshold for abnormality does not exist. The demonstration that voltage-guided ablation using 0.5 mV as a cut-off can result in improved clinical outcomes does nevertheless add credibility to this figure as a useful clinical threshold for detecting abnormal tissue, but it does not refute that there is potentially a better way of defining abnormal tissue.

### Voltage mapping and rhythm

Atrial rhythm appears to be an important determinant of bipolar voltage. Ndrepepa et al. found that voltages in the left and right atria were lower during AF than during sinus rhythm [67]. Moreover, sites with the greatest difference in voltage showed the shortest AF cycle length suggesting an effect from more disorganised, rapid atrial depolarisation or depolarisation occurring through partially depolarised tissue [67]. In addition, there is evidence to suggest that low voltage sites during AF may show normal voltages during coronary sinus pacing [13, 66]. Furthermore, in patients with both AF and atrial flutter, Bradfield et al. demonstrated higher voltages while mapping in atrial flutter than in sinus rhythm [68], supporting a hypothesis that tachycardia itself is not wholly responsible for lowering atrial voltage but that continually changing wave-front activation direction during AF also contributes [69].

Yagishita et al. demonstrated a modest linear correlation between voltages obtained in sinus rhythm and AF, but the relationship was weaker in patients with persistent than paroxysmal AF (*r* = 0.53 vs. *r* = 0.718). A possible explanation for this discrepancy may be increased anisotropy in patients with persistent AF leading to lower voltages. Additionally, significantly more low voltage areas were identified when mapping was performed in AF than in sinus rhythm [70].

Despite the marked differences in atrial voltage during different rhythms, there is no consensus for which rhythm is most appropriate for substrate mapping. There is significant inter-operator variation throughout the literature with regard to the rhythm, pacing site and activation rate at which voltage maps are created. Chang et al. tried to compensate for differences in voltage recorded in different rhythms by using the root mean square of the AF voltage, a metric which compensates for variation in amplitude during AF over time, derived from unipolar electrograms. They showed an improved correlation between sinus rhythm voltage and root mean square AF voltage compared to peak negative unipolar voltage [71], suggesting that this method can reduce the overall effect of changes in rhythm. Nevertheless, it is clear that the lack of standardisation can create variation in voltage maps. In our institution, all voltage maps are therefore created during coronary sinus pacing at either 500 or 600 ms (depending on the sinus rate) to reduce such variation.

### Regional changes in voltage

Several studies have shown variation in voltages between atrial regions. For example, in a study of 20 patients with AF, the highest voltage was found in the left atrial floor and the lowest in the left atrium–pulmonary vein junctions [62]. Marcus et al. compared regional voltage in AF patients to those with atrial tachycardia and found voltage in AF patients was 0.8 mV lower at the posterior wall and 0.6 mV lower at the septum, compared to the anterior wall [19]. A further study found the posterior wall to have higher voltages than other segments of the left atrium when mapping in AF [72], whereas another has found the appendage and mid anterior and posterior wall to have the highest voltages [73]. More recently, Kogawa et al. have shown that regional differences exist between paroxysmal and persistent AF patients [74]. Given that voltage is affected by atrial wall thickness [75] and that wall thickness varies through the atria [76], it is likely that some of these differences are explained by variations in wall thickness; however, other factors such as external compression [77] and regional atrial wall stress may also influence recorded voltages [39]. Currently, no accommodation is made during mapping for the likely different normal values for different regions of the atria.

## New approaches to atrial voltage mapping

Two new techniques may be able to improve the detection of abnormal atrial substrate using electrogram amplitude characteristics.

### Omnipole mapping

Omnipolar mapping is a recently developed technology for recording electrograms which is theoretically insensitive to catheter orientation. The technology simultaneously records unipolar electrograms from a multi-electrode catheter that spans 3D space to derive conduction velocity and wave-front direction [78]. Recent animal and computer models have shown an excellent correlation between omnipolar and optical mapping signals with regard to both conduction velocity and activation direction [79]. By comparing different orientations of bipolar electrograms from the electrode grid, omnipolar electrograms match those of the largest bipolar electrogram, removing the influence of reduced amplitude due to activation direction. This relative increase in electrogram amplitude has been shown to change the voltage threshold by which tissue can be histologically defined as ventricular scar (1.5 mV), far higher than the standard 0.5 mV for conventional bipolar mapping [80]. In addition, the size of ablation lesions appears overestimated using bipolar rather than omnipolar electrograms, in line with recent data from our group [81].

Most recently, Haldar et al. used omnipolar mapping to derive larger and more consistent electrogram amplitudes within the canine atrium during AF. They showed significant differences between mean voltage amplitude of horizontal and vertically orientated bipoles in sinus rhythm, but not during AF [82]. As a result significantly different scar maps were created depending on whether electrodes were horizontally or vertically placed. Currently, no published data in human atrial fibrillation exists where omnipolar mapping theoretically overcomes a significant limitation of bipolar voltage mapping. Further work will be necessary to determine whether stronger associations between voltage and other measures of atrial fibrosis such as late gadolinium enhancement CMR are identifiable when omnipolar rather than bipolar voltage mapping is employed.

### Dynamic voltage mapping

Several pieces of evidence indicate that lower voltage is linked to shorter AF cycle lengths and that atrial voltage may be dependent on activation rate. For example, one study has shown left atrial voltage was lower when pacing from the coronary sinus (at 50 ms faster than the sinus rate) than during sinus rhythm. Although this difference was seen in both AF and non-AF patients, the decrease in voltage was more pronounced in AF patients [83]*.* Further, increasing coronary sinus activation rate has been shown to increase the size of low voltage areas in patients with paroxysmal AF [84]. In addition, pacing with two different cycle lengths (600 ms and 300 ms) and mapping with a 64-pole basket catheter has shown reduced voltage at the faster pacing rate at sites with voltage > 1.5 mV during sinus rhythm, but no significant difference at sites between 0.5 mV and 1.5 mV [85].

**Fig. 5:**
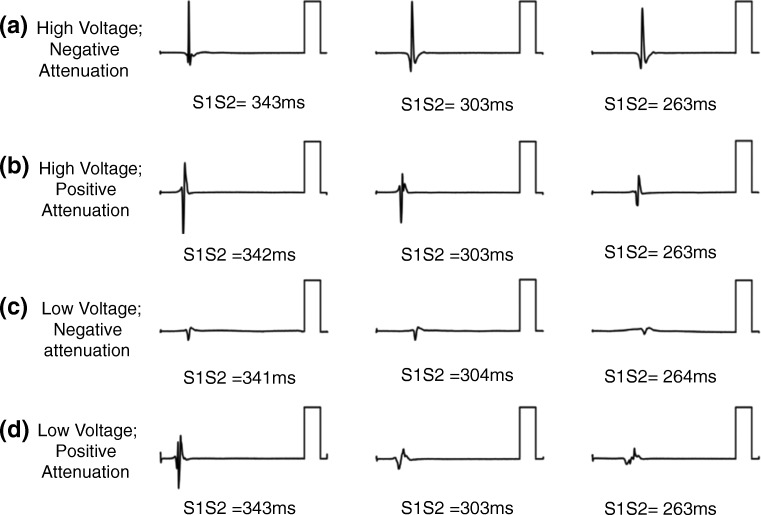
Bipolar voltage attenuation at decreasing coupling intervals: Four unique patterns found when measuring voltage amplitude during decreasing coupling intervals in patients with otherwise ‘normal’ voltage (> 0.3 mV at CL 470 ms). (**a**) High initial voltage without attenuation. (**b**) Voltage with attenuation that plateaus above 1 mV. (**c**) Initial low voltage without attenuation. (**d**) Voltage which attenuates to a low plateau (< 1 mV) [101]

Based on these observations, we recently performed extra-stimulus testing at decreasing coupling intervals from the coronary sinus in paroxysmal AF patients without low voltage areas. We found that left atrial voltage attenuated with decreasing coupling intervals at over two-thirds of atrial sites. In our study, we split these sites into those at which the lowest voltage achieved was greater or less than 1 mV. In the remaining 30% of sites, either high (> 1 mV) or low (< 1 mV) voltage was recorded without any significant change in voltage with activation rate (Fig. 5). Both unipolar electrogram voltage and conduction velocity were significantly reduced at sites showing voltage attenuation therefore potentially identifying candidate sites important for the maintenance of AF. These data revealed significant electrical heterogeneity between AF patients which was undetectable by standard voltage mapping suggesting that static voltage mapping alone may be insufficient to accurately characterise the AF substrate. A key advantage of dynamic voltage mapping is that change in voltage from baseline (i.e. change with activation rate) may be less sensitive to the sources of error influencing bipolar voltage mapping outlined in Section 3. Further studies assessing dynamic voltage correlation with cardiac magnetic resonance imaging are planned to determine if this technique is indeed more accurate than standard voltage mapping to detect atrial fibrosis.

## Conclusions

Voltage mapping is a straightforward procedure for the skilled electrophysiologist, providing rapid and seemingly intuitive data. It is in part for these reasons that voltage mapping has become the current gold standard marker for atrial fibrosis and voltage-guided ablation has shown initial promise for the treatment of atrial fibrillation. The amplitude of the bipolar electrogram represents a rich data source when recorded under standardised conditions; however, uncontrolled variables such as activation direction, catheter incident angle and atrial tissue factors, such as conduction velocity and tissue thickness, raise valid concerns regarding the accuracy of voltage mapping to detect and quantify atrial fibrosis. Furthermore, the exact nature of the threshold between histological fibrosis and low voltage has not been described. Indeed, the finding of dynamic voltage attenuation suggests that a single binary threshold may not exist which can accurately characterise atrial fibrosis. It is as yet unclear to what extent these sources of variability could influence patient outcomes with the increasing uptake of ablation strategies targeting low voltage areas.
